# Negative Myoclonus Secondary to Thalamic Infarction: Case Report

**DOI:** 10.5334/tohm.629

**Published:** 2021-06-29

**Authors:** Talita Aparecida Conte, Leo Coutinho, Hélio A. Ghizoni Teive

**Affiliations:** 1 Neurology Service, Internal Medicine Department, Hospital de Clínicas, Federal University of Paraná, Curitiba, Paraná, Brazil; 2 Movement Disorders Unit, Neurology Service, Internal Medicine Department, Hospital de Clínicas, Federal University of Paraná, Curitiba, Paraná, Brazil; 3 Neurological Diseases Group, Graduate Program of Internal Medicine, Internal Medicine Department, Hospital de Clínicas, Federal University of Paraná, Curitiba, Paraná, Brazil

**Keywords:** Movement disorders, Hyperkinesias, Myoclonus, Thalamus, Stroke

## Abstract

**Background:**

Movement disorders are an infrequent presentation to stroke, and in this context, negative myoclonus is not among the most common movement disorders, hence we present a case of negative myoclonus secondary to thalamic stroke.

**Case:**

A 75 year old male presented with left central facial palsy and negative myoclonus on his left upper limb. He was submitted to a diagnostic workup, with evidence of a right thalamic stroke. He was started on Phenobarbital 50 mg and the movement disorder resolved.

**Conclusion:**

Given the relevance of cerebrovascular disease as a cause of morbidity and mortality, it is important to the clinician to be aware of the less typical presentations such as the observed in our case, in order to provide adequate care to the patient.

## Introduction

Negative myoclonus consists of a sudden, brief, arrhythmic lapse of sustained posture caused by an involuntary interruption of muscle contraction. It is commonly described in the context of hepatic encephalopathy, but is also related to other toxic-metabolic etiologies, as well as structural causes. We hereby describe an unusual presentation of thalamic stroke, featuring negative myoclonus [1].

## Case Report

**Figure 1:**
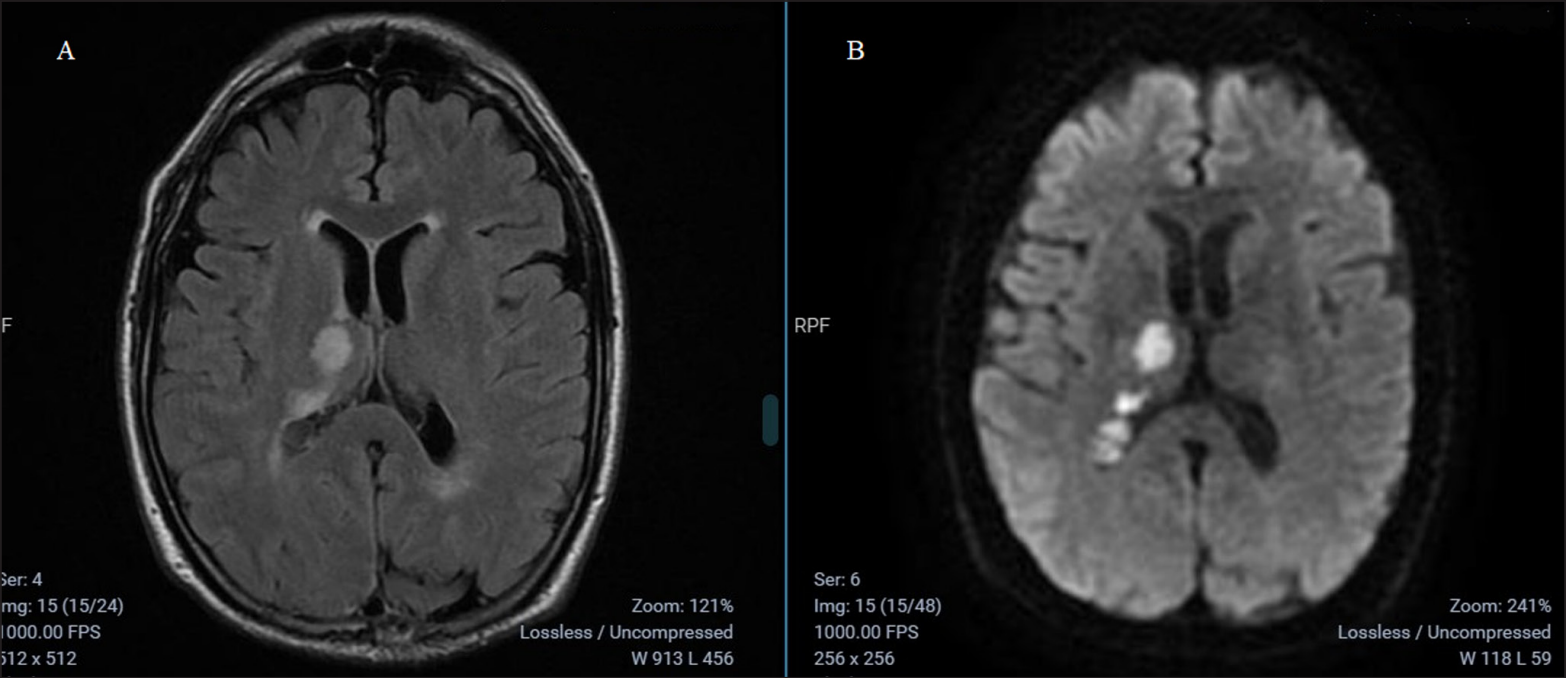
**MRI images obtained on the 2^nd^ day of admission**. MRI images showing signs of acute vascular insult on the right thalamus, marked by T2/FLAIR hyperintensities **(A)** and restricted water diffusion on DWI **(B)**.

A 75 year old male patient presented to the emergency room, complaining of a sudden headache, accompanied by vomiting and a left central facial palsy. He was previously diabetic and hypertensive, with irregular treatment. He also had a prior history of stroke, 4 years ago, with no functional sequel. His neurological examination showed, apart from the facial palsy, a negative myoclonus at his left upper limb (***Video 1***), and mild left dysmetria at index-nose test, with the remaining of the examination unremarkable. He was submitted to a non-contrast CT scan and a CTA, suggesting an acute posterior circulation stroke, with an occlusion of the P2 segment, and severe stenosis of the P1 segment of the right posterior cerebral artery (PCA). Given the initial evaluation suggesting minor stroke, featuring ASPECTS of 10 and NIHSS of 3, intravenous thrombolysis was not indicated. An MRI study held 2 days after the ictus showed marked T2/FLAIR hyperintensities at the right temporal and occipital lobes, in addition to the ipsilateral caudate nucleus and thalamus, with restricted water diffusion on DWI (***Figure 1***). He was also submitted to an EEG, with no epileptiform activity. A toxic-metabolic panel was performed, with normal CBC, electrolytes, renal function and vitamin B12. Ancillary investigation also included serology testing for HIV, Hepatitis B/C and Syphilis, all negative. CSF samples were obtained, with no alterations to cellularity, biochemical analysis, culture and opening pressure. As for the myoclonus, the patient was started on Phenobarbital 50 mg a day, resulting in amelioration of the movement disorder. He received dual antiplatelet therapy and was discharged in his fourth day of admission, after a complete stroke workup that showed no remarkable features apart from the PCA stenosis. Patient received orientations concerning careful tapering of Phenobarbital.

**Video 1:** **Physical examination on admission**. The patient presented with sudden, arrhythmic movements with intermittent loss of postural tone on his left upper limb, compatible with negative myoclonus. The second segment of the video (between 12 and 17 seconds) shows partial improvement of the myoclonus after administration of Phenobarbital 50 mg. The abnormal movements were later completely suppressed.

## Discussion

Negative myoclonus, or asterixis, is a movement disorder characterized by spontaneous, involuntary, intermittent loss of muscular postural tone, usually bilateral. Unilateral cases can occur in approximately 1,9% of post-stroke lesions, typically associated to contralateral thalamic involvement, in particular the ventrolateral and ventroposterior nuclei. Less often, this event can be related to structural lesions in the frontal lobe, lenticular nucleus, internal capsule, precentral cortex, midbrain or cerebellum [1,2].

On occasion, focal unilateral lesions can cause bilateral negative myoclonus, for example in lesions determining mass effect. Although there are reports of minor structural lesions determining ipsilateral myoclonus, the common occurrence of metabolic comorbid conditions in these case reports cause confusion biases, preventing the establishment of causative relation [1].

A recent systematic review on post-thalamic stroke movement disorders, featuring data from 86 papers, showed 37 patients with negative myoclonus, among them 11 cases with associated hemiataxia and one case with associated dystonia. Most of the cases resolved spontaneously within a period of 10 days. The most common thalamic nucleus involved was the ventrolateral nucleus [3].

Postural control is maintained by a complex interplay between multiple pathways, such as the vestibulospinal, reticulospinal and rubrospinal tracts, regulated by supratentorial structures, particularly the ventrolateral nucleus of the thalamus, the prefrontal area and medial frontal cortex. The apparent causative mechanism of negative myoclonus in stroke is an involvement of these cerebello-rubro-thalamo-cortical projections, causing the intermittent loss of postural control, and thus determining the myoclonus [3,4,5]. The occurrence of ipsilateral upper limb ataxia in our case suggests a similar involvement of these projections.

Another account of post-stroke movement disorders, comprising 284 published cases, showed myoclonus as the fourth most common movement disorder in their cohort, after dystonia, tremor and chorea. However, this study did not differentiate among asterixis and other forms of myoclonus. In this paper the frontal lobe lesions were the leading causes of myoclonus, and 83,7% of cases resolved spontaneously [6].

As for the treatment, our option on utilizing Phenobarbital resided on its omnipresence in the Brazilian public health facilities, as opposing to the first-line agents typically prescribed to symptomatic treatment of myoclonus, such as Levetiracetam, Valproic Acid and Clonazepam, much less accessible in the Brazilian public health system [7]. The reasonable effectiveness of Phenobarbital to treat myoclonus, its adequate security profile and low drug to drug interaction level were other factors held in account for our selection of the given anticonvulsant.

Our report possesses a few limitations. Ideally, the myoclonic movements should have been evaluated through electroneuromyography. An eletrophisiologic assessment could help to better characterize the negative myoclonus, its frequency and duration, as well as determine overlapping of positive myoclonic movements. Another shortcoming of our case was the absence of video documentation of finger-nose examination and the upper limb ataxia as well as comparison to the right upper limb.
